# Coats-like Vasculopathy in Inherited Retinal Disease: Prevalence, Characteristics, Genetics, and Management

**DOI:** 10.1016/j.ophtha.2023.07.027

**Published:** 2023-12

**Authors:** Malena Daich Varela, Giovanni Marco Conti, Samantha Malka, Veronika Vaclavik, Omar A. Mahroo, Andrew R. Webster, Viet Tran, Michel Michaelides

**Affiliations:** 1 Moorfields Eye Hospital, London, United Kingdom; 2 UCL Institute of Ophthalmology, University College London, London, United Kingdom; 3 Hôpital Ophtalmique Jules-Gonin, Lausanne, Switzerland

**Keywords:** Coats, Inherited, Genetics, Retina, Vasculopathy

## Abstract

**Purpose:**

To describe the largest, most phenotypically and genetically diverse cohort of patients with inherited retinal disease (IRD)-related Coats-like vasculopathy (CLV).

**Design:**

Multicenter retrospective cohort study.

**Participants:**

A total of 67 patients with IRD-related CLV.

**Methods:**

Review of clinical notes, ophthalmic imaging, and molecular diagnosis from 2 international centers.

**Main Outcome Measures:**

Visual function, retinal imaging, management, and response to treatment were evaluated and correlated.

**Results:**

The prevalence of IRD-related CLV was 0.5%; 54% of patients had isolated retinitis pigmentosa (RP), 21% had early-onset severe retinal dystrophy, and less frequent presentations were syndromic RP, sector RP, cone-rod dystrophy, achromatopsia, *PAX6*-related dystrophy, and X-linked retinoschisis. The overall age of patients at CLV diagnosis was 30.7 ± 16.9 years (1–83). Twenty-one patients (31%) had unilateral CLV, and the most common retinal features were telangiectasia, exudates, and exudative retinal detachment (ERD) affecting the inferior and temporal retina. Macular edema/schisis was observed in 26% of the eyes, and ERD was observed in 63% of the eyes. Fifty-four patients (81%) had genetic testing, 40 of whom were molecularly solved. Sixty-six eyes (58%) were observed, 17 eyes (15%) were treated with a single modality, and 30 eyes (27%) had a combined approach. Thirty-five eyes (31%) were “good responders,” 42 eyes (37%) were “poor responders,” 22 eyes (19%) had low vision at baseline and were only observed, and 12 eyes (11%) did not have longitudinal assessment. Twenty-one observed eyes (62%) responded well versus 14 (33%) treated eyes. Final best-corrected visual acuity was significantly worse than baseline (*P* = 0.002); 40 patients (60%) lost 15 ETDRS letters or more over follow-up in 1 or both eyes, and 21 patients (31%) progressed to more advanced stages of visual impairment.

**Conclusions:**

Inherited retinal disease–related CLV is rare, sporadic, and mostly bilateral; there is no gender predominance, and it can occur in diverse types of IRD at any point of the disease, with a mean onset in the fourth decade of life. Patients with IRD-related CLV who have decreased initial visual acuity, ERD, CLV changes affecting 2 or more retinal quadrants, and *CRB1-*retinopathy may be at higher risk of a poor prognosis.

**Financial Disclosure(s):**

Proprietary or commercial disclosure may be found in the Footnotes and Disclosures at the end of this article.

Coats disease (CD) is a rare ocular disorder characterized by abnormal retinal vasculature, manifesting as telangiectasia, aneurysms, hemorrhages, or intraretinal and subretinal exudation.^1^ It has a prevalence of approximately 0.025% and it is an idiopathic, isolated disorder. However, Coats-like vasculopathy (CLV) can appear associated with systemic diseases such as facioscapulohumeral muscular dystrophy or rare genetic syndromes, including syndromic or non-syndromic retinal diseases.^2^, ^3^, ^4^ Both are believed to occur due to abnormal endothelium and pericytes that lead to defects in the blood-retinal barrier, exudation, vessel tortuosity and dilation, retinal ischemia, and detachment.^5^^,^^6^

Coats disease is typically described as a unilateral condition, affecting more male than female patients in late childhood or adolescence.^4^^,^^7^ Patients often have decreased visual acuity and less frequently strabismus, pain, and xanthocoria.^3^ Coats disease can be classified according to the severity of its presentation, ranging from telangiectasia only to total retinal detachment (RD) and glaucoma.^8^ Similar to other retinal vasculopathies (i.e., diabetic retinopathy and retinal vein occlusion), CD initially develops in the retina, but it can affect the eye as a whole in advanced disease stages, when ischemia-led neovascularization compromises the ciliary angle and iris, and intraocular pressure rises.^8^ There are several approaches to CD management depending on the severity of the presentation; observation, laser photocoagulation, cryotherapy, anti-VEGF, depot steroids, and vitrectomy are current available options.^9^

Inherited retinal diseases (IRDs) are a diverse group of mostly monogenic conditions that commonly manifest as a degeneration of photoreceptors and retinal pigment epithelium cells. They include diverse phenotypes, such as early-onset severe retinal dystrophy (EOSRD), rod-cone dystrophy (also known as “retinitis pigmentosa” [RP], the most common type), cone-rod dystrophy (CORD), isolated cone, and macular dystrophies, among others.^10^ Approximately 1 in 2000 individuals are affected worldwide, and the age of onset is widely variable, comprising congenital to late-adulthood disorders.^11^

The first case of CLV affecting a patient with an IRD was described in 1956 in an individual with RP.^12^ Since then, multiple case reports have described patients with RP and CLV retinal changes, and it is estimated that the latter affect 1% to 5% of patients with RP.^13^, ^14^, ^15^ Previous studies have characterized RP-related CLV in smaller cohorts, with a focus on *CRB1*-associated IRD.^15^, ^16^, ^17^, ^18^ In this study, we describe the largest cohort of patients with IRD-related CLV to date and discuss its management, prognosis, and genotype-phenotype correlations.

## Methods

This study was a retrospective case series of patients who attended Moorfields Eye Hospital (London, UK) and Hôpital Ophtalmique Jules-Gonin (Lausanne, Switzerland) and were diagnosed with an IRD and CLV changes. Patients were identified through a clinical database search. Some of the terms queried were “pigmentosa,” “exudative,” “dystrophy,” “exudates,” “vasoproliferative,” “telangiectasia,” “coat,” “coats,” “coats-like,” “degeneration,” “inherited,” “Leber,” “vasculopathy,” and “proliferative.” Informed consent was obtained from all patients. Ethical approval was provided by the local ethics committee, and the study honored the tenets of the Declaration of Helsinki.

Patient electronic healthcare records and paper notes were reviewed to retrieve relevant clinical information. The diagnosis of IRD was made by retinal examination, ocular, medical and family history, multimodal retinal imaging, and when available, electroretinography and genetic testing. All patients had an unremarkable birth history and were born full-term.

Age of disease onset corresponds to the age at which visual symptoms were first noticed by the patients or caregivers. Snellen visual acuities were converted to logarithm of the minimum angle of resolution (logMAR) for statistical analysis purposes. As per conventions,^19^^,^^20^ count fingers vision was given a value of logMAR 1.98, hand motion vision was given a value of logMAR 2.28, light perception vision was given a value of logMAR 2.7, and no light perception vision was given a value of logMAR 3.0. Patients were categorized using the World Health Organization (WHO) visual impairment criteria that define no or mild visual impairment as best-corrected visual acuity (BCVA) < 0.48 (6/18, 20/60), moderate impairment as BCVA > 0.48 and < 1.0 (6/60, 20/200), severe impairment as BCVA > 1.0 and < 1.3 (3/60, 20/400), and blindness as BCVA > 1.3.

Ultra-widefield pseudocolor, autofluorescence (green), and fluorescein angiogram imaging were taken with Optos (Optos PLC, Dunfermline, UK), and macular optical coherence tomography (OCT) scans were obtained with Spectralis (Heidelberg Spectralis, Heidelberg Engineering, Inc., Heidelberg, Germany). Genetic testing was performed using panel-based, targeted next-generation sequencing, whole exome sequencing, or whole genome sequencing. When appropriate and available, parental blood samples were taken to confirm segregation of proposed variants. The pathogenicity of previously unreported variants was determined by implementing the criteria of the American College of Medical Genetics.^21^^,^^22^

GraphPad Prism 8.0.2 (GraphPad Software) was used for statistical analysis. The threshold of significance was set at *P* < 0.05. *T* tests and linear regressions were used to assess parametric variables. Welch's *t* test variation was used when the sample sizes were significantly different.

## Results

### Prevalence, Demographics, Phenotype, and Disease Onset

Two patients had IRD-related CLV among 890 patients with IRD in the Swiss center and 65 among approximately 10 000 patients with IRD at Moorfields Eye Hospital, resulting in a prevalence of approximately 0.5% of CLV in patients with IRD.

Sixty-seven patients from 67 families were included in this study (Table S1, available at www.aaojournal.org). Ethnicity was available in 47 patients (70%); 26 patients (39%) were White, 14 patients (21%) were Asian, 6 patients (9%) were Black, and 1 patient (1%) was of mixed race. Thirty-one patients were male (46%), and 36 patients were female (54%). Positive family history was reported by 29 patients (43%), and 12 patients mentioned consanguinity in their family (18%), with parents being first cousins.

Thirty-six patients had RP (54%, 11 having dominant RP; 23 having recessive RP; and 2 having X-linked RP), 14 patients (21%) had EOSRD, 10 patients had syndromic RP (15%, 2 had Usher syndrome [USH] type 1, 5 had USH type 2, 1 had Bardet-Biedl syndrome, 1 had Hallervorden-Spatz disease, and 1 had unknown etiology), 2 patients (3%) had sector RP, 2 patients had CORD, 1 patient had achromatopsia, 1 patient had *PAX6*-related dystrophy, and 1 patient had X-linked retinoschisis.

The age of visual symptoms onset was 12.2 ± 12 years (mean ± standard deviation; median, 8, range, birth to 55 years), and the mean age of IRD diagnosis was 22.0 ± 17.1 years (median, 16 years, range, 2–61 years). Age at baseline visit was 27.5 ± 16.4 years (median, 26 years). Eighteen patients were children (27%, age < 16 years), and 49 patients (73%) were adults.

Forty patients (60%) were found to have CLV and IRD at their first visit, 25 patients (37%) developed CLV after 8.3 ± 8.6 years of follow-up (median 5, 0.5–39), and 2 patients (3%) were referred with a diagnosis of CLV and were subsequently found to have an IRD. The overall age of CLV diagnosis was 30.7 ± 16.9 years (median, 29 years, 1–83 years).

### Visual Acuity

Initial BCVA of those without CLV changes at baseline (n = 26, 26.1 ± 18.6 years) was 0.6 ± 0.7 in the right eye (OD) and 0.9 ± 0.9 in the left eye (OS), whereas those with CLV at baseline had 1.1 ± 0.9 in the OD and 1 ± 1 in the OS (n = 41, 29.9 ± 14.7 years). There was a significant difference for the OD (*P* = 0.01), but not for the OS (0.87). Baseline age was not significantly different between those with and without CLV changes (*P* = 0.5).

At baseline, 36 patients had no to mild visual impairment (14 without CLV, age 9–78 years), 20 patients had moderate visual impairment (7 without CLV, 8–42), 2 patients had severe visual impairment (a 5-year-old without CLV and a 12-year-old), 8 patients were blind (2 without CLV, 11–42), and 1 was an infant. Asymmetric BCVA (difference < 0.3) at the initial visit was seen in 20 patients with CLV (50%) and 7 patients without CLV (28%). Thirty-nine eyes with CLV (35%) had low vision (BCVA > 1.3) when CLV was first noticed.

There was no significant association between age and BCVA in patients with CLV at baseline (*P* = 0.3) or patients without CLV (*P* = 0.1) when analyzed cross-sectionally at their initial visit.

Those with CLV development over follow-up had a BCVA of 1.3 ± 1 in the OD and 1.1 ± 1 at the time of CLV onset (age 33.2 ± 19.8 years). Considering the affected eyes only, there was a significant difference between BCVA at baseline versus at CLV onset (*P* = 0.0003).

Forty-four eyes (39%) had CLV changes affecting the macula, 51 (45%) had them peripherally, and in 18 eyes (16%) this was unclear due to advanced disease, history of CLV complications at baseline, or poor visibility of the posterior pole. Baseline BCVA for those with CLV affecting the macula was 1.2 ± 0.8 OD and OS versus 0.5 ± 0.6 OD and 0.6 ± 0.8 OS in those with the periphery affected only (significantly different, *P* = 0.005 OD and 0.05 OS).

### Clinical Examination, Fundus Imaging, and Fluorescein Angiography

Forty-nine patients (73%) developed lens opacities at a mean age of 34.6 ± 15.7 years, from congenital to 69 years of age.

**Figure 1:**
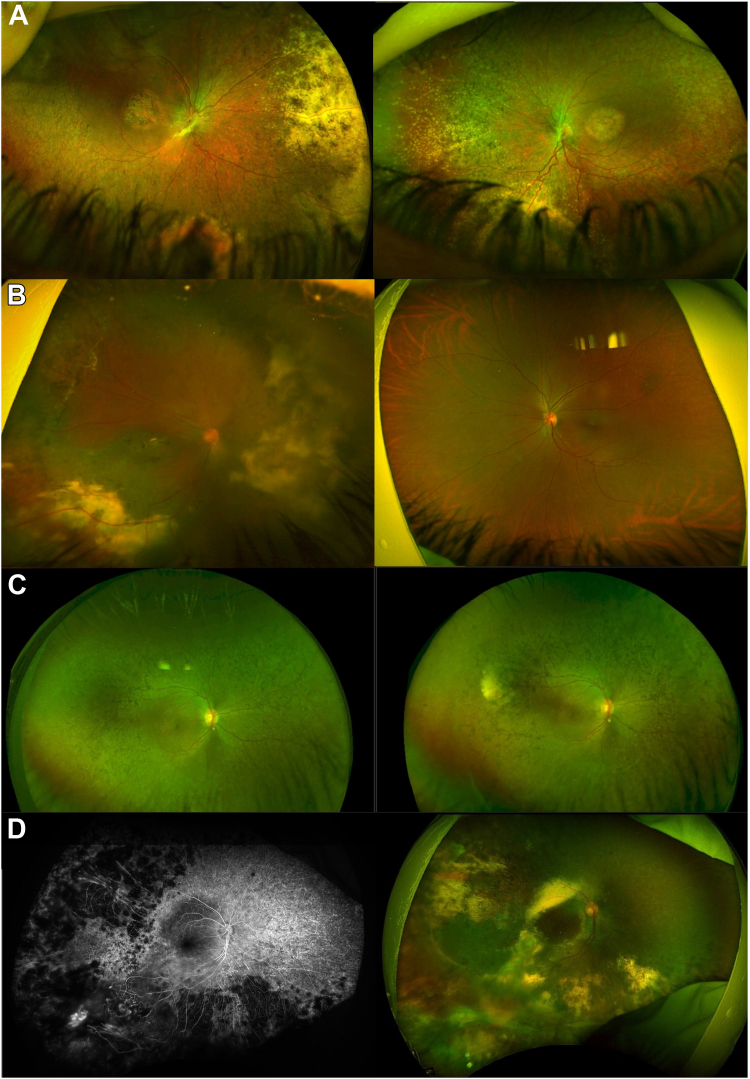
Fundus features of Coats-like vasculopathy (CLV) in inherited retinal disease (IRD). **A,** Patient ID 59 at 7 years of age. A retinal dystrophy affecting both the macula and peripheral retina is evident, with bilateral exudation in the nasal and inferior retina. This patient had negative genetic testing results and a syndromic IRD with developmental delay, maxillary and mandibular hypoplasia, and pontocerebellar atrophy. **B,** Patient ID 2 with *GNAT2*-related achromatopsia at 35 years of age. Peripheral atrophy with exudation is visible unilaterally in the right eye (OD). **C,** The OD of patient ID 13 at 13 and 18 years of age. We see the development of retinal exudation and telangiectasia over time in the superotemporal quadrant. She had negative genetic testing and a more severely affected left eye, with recurrent vitreous hemorrhages. **D,** Fluorescein angiogram and color retinal imaging of the OD of patient ID 7 at 15 years old. Temporal nonperfusion and telangiectasia are visible. Color imaging is positive for retinal exudation and atrophy in 3 retinal quadrants. Genetic testing was not performed.

Sixty-four eyes (57%) had macular OCT scans, and 29 eyes (26%) had macular edema/schisis during follow-up. All patients had varied degrees of retinal pigmentary changes, vessel thinning, optic disc pallor, and macular involvement (Fig 1A and B).

Twenty-one patients had unilateral CLV changes (31%, 15 OD and 6 OS) and 46 patients had bilateral, asymmetric disease (69%), giving a total of 113 eyes affected with CLV and IRD. The maximum time until the contralateral eye showed CLV signs was 7 years (Fig 1C). Twenty eyes (18%) had telangiectasia only, 6 eyes (5%) had exudative RD only, 1 eye (1%) had retinal exudation only, 2 eyes had a vasoproliferative tumor only (2%), and the remaining 84 eyes (74%) had a combination of the previous angiomatous malformations, retinal exudates, and hemorrhages. Fifty-four eyes (48%) had up to 2 quadrants affected (mostly inferior and temporal), and 59 eyes (52%) had 3 or more quadrants involved.

Seventy-one eyes (63%) developed exudative RD at or after 1.3 ± 4.3 years of CLV diagnosis (0–20): 6 total RD, 26 inferior, 5 posterior, 4 temporal, and the remaining eyes had a combined location (e.g., inferotemporal, inferior, and posterior).

**Figure 2:**
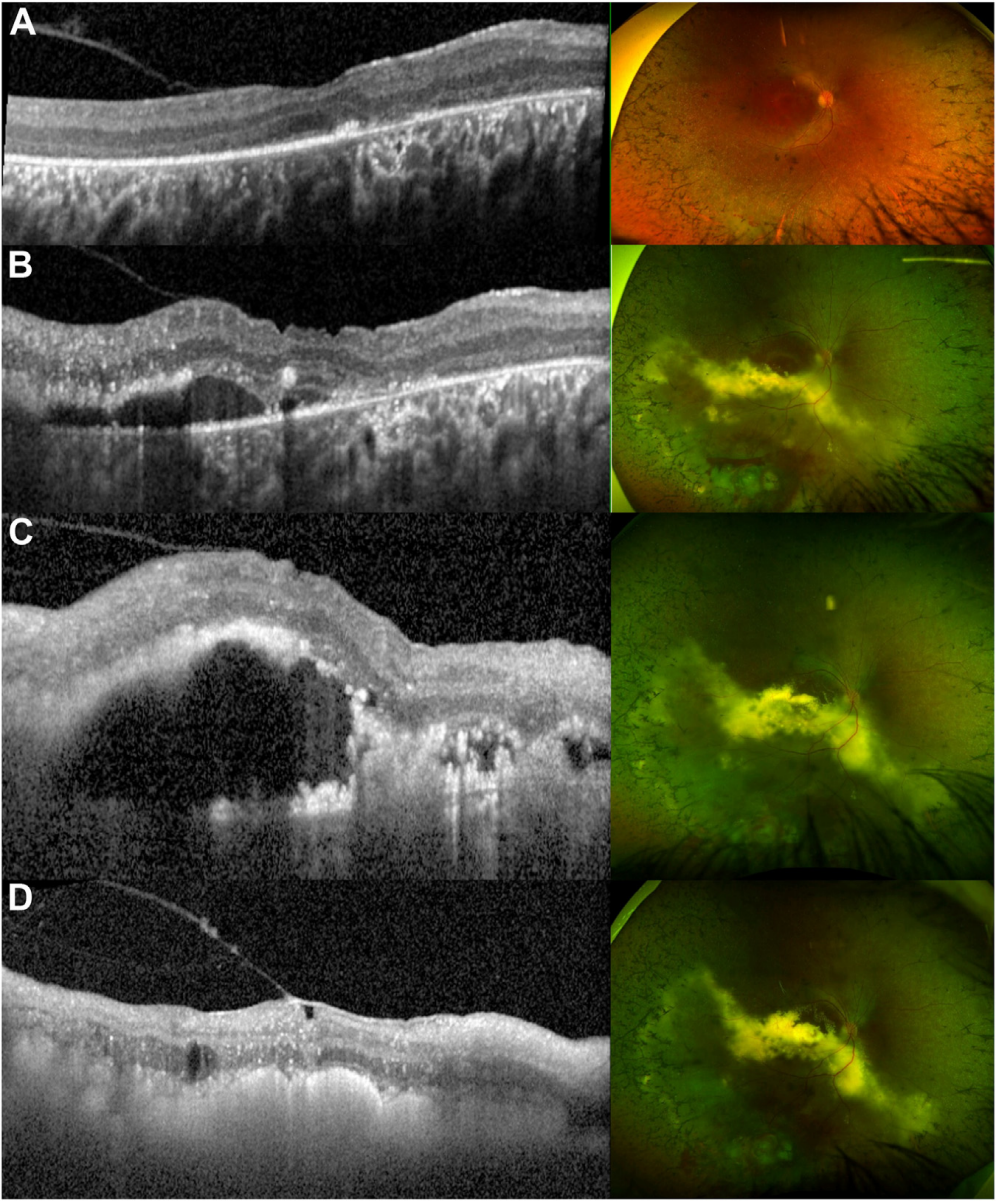
Macular OCT and ultrawide-field color fundus imaging of a patient with *RPE65* early-onset severe retinal dystrophy and Coats-like vasculopathy (CLV) (ID 60) before and after treatment. **A,** Two years before the appearance of CLV changes, the scan is characterized by an atrophic outer retina and abnormal vitreoretinal interface. There are no signs of CLV in fundus imaging. **B,** Scan taken when patient came to the emergency service due to sudden unilateral decrease in visual acuity, subretinal fluid is visible in OCT and telangiectasia, exudative retinal detachment (ERD), and retinal hemorrhages are seen in fundus evaluation and imaging. Cryotherapy and anti-VEGF treatment were scheduled. **C,** Scan and image taken 1 month after treatment, subretinal fluid and exudation are increased, and a second anti-VEGF was planned. **D,** Scan and image taken 1 month after the second anti-VEGF injection. The subretinal fluid has resolved, and retinal atrophy, intraretinal cysts, and a thickened epiretinal membrane are visible.

Fifteen patients had fluorescein angiography (22%); 9 patients had poor peripheral perfusion, 6 patients showed neovascularization with leakage, and 1 patient had a vasoproliferative tumor-like mass (Fig 1D). Patient ID 60 had participated in an *RPE65* gene supplementation clinical trial (NCT02781480) 7 years before the development of CLV in the treated eye, noticed due to an acute drop in vision (Fig 2). He received 0.4 ml of low-dose AAV-OPTIRPE65 in the OD. His overall response to the intervention had been positive until this episode, with no complications. He subsequently received Luxturna in the contralateral eye 4 years after the first surgery, and fundus examination revealed no CLV until his most recent visit.

### Management and Longitudinal Analysis

Sixty patients (90%) had longitudinal assessments, with a mean follow-up time of 15.6 ± 13.1 years (0–57) and mean age of 44.4 ± 20.3 years (median, 43) at the latest visit. Final BCVA was 1.8 ± 1 logMAR OD and 1.5 ± 1.1 OS, with a significant difference between baseline and follow-up BCVA (*P* = 0.002). A significant difference was also found in final BCVA between patients with macular and peripheral CLV (*P* < 0.0001 and 0.0003).

Forty patients (60%) lost 15 ETDRS letters or more over follow-up in 1 or both eyes. Twenty-one patients (31%) progressed to more advanced WHO categories of visual impairment over follow-up, 15 of whom became blind (22%, age 10–75 years).

Sixty-six eyes (58%) were observed, 9 (8%) had laser treatment only, 5 (4%) had cryotherapy only, 2 (2%) had pars plana vitrectomy (PPV), 1 (1%) had a scleral thinning and draining procedure, and 30 (27%) had a combined approach. Eyes that maintained BCVA < 1.3 at the latest assessment were classified as “good responders,” and those that received treatment and had a final BCVA > 1.3 were labeled as “poor responders.”

#### Observation Only

This group had a baseline age of 30.3 ± 19 years, follow-up time was 16 ± 14.2 years, and the age at the most recent assessment was 45.6 ± 21.6 years.

Twenty-two eyes had low vision at baseline (33%, BCVA >1.3), 21 eyes (32%) were good responders, 13 eyes (20%) were poor responders, 9 eyes (14%) did not have follow-up assessments, and 1 patient was nonverbal (2%, Table S2, available at www.aaojournal.org). The eyes that did not respond well to observation were left untreated for reasons such as older cases where minimally invasive intravitreal medication was not approved/available yet, pregnant/nursing patients, functioning contralateral eye, and patient’s choice.

The complications seen in this subgroup were exudative RD (33 eyes), vitreous hemorrhage (VH) (7 eyes), intraretinal or retrohyaloid hemorrhages (3 eyes), neovascular glaucoma (5 eyes), open-angle glaucoma (4 eyes), acute angle-closure glaucoma (2 eyes), band keratopathy (2 eyes), and ocular inflammation (episcleritis and anterior uveitis, 2 eyes; Table S2).

#### One Therapeutic Method Only

Patients treated solely with laser, cryotherapy, PPV, or a scleral thinning and draining procedure were combined for analysis (n = 17). Their age at baseline was 27.9 ± 11.6 years, they were followed for 18.1 ± 13.9 years, and their age at the latest assessment was 48.8 ± 18.4 years. Four eyes had serial procedures. Nine eyes had between 1 and 5 sessions of focal or panretinal photocoagulation; 4 eyes had only 1 session of cryotherapy, and the remaining eye had 3 sessions; 1 eye had 2 PPVs, 1 eye had 1 PPV, and the remaining eye had 1 scleral thinning procedure with drainage of subretinal fluid.

Ten eyes (59%) had poor response to treatment, with decreased vision, development of exudative and/or tractional RD (10 eyes), glaucoma (6 eyes, neovascular and/or acute angle-closure glaucoma), VH (5 eyes), and chronic uveitis post-treatment (1 eye). Four eyes (24%) had good response, with resolved RD, inactive telangiectasia, and absorbed hemorrhages; 2 eyes (12%) did not have follow-up assessments, and 1 patient (6%) was nonverbal (Table S2).

#### Combined therapy

Patients treated with more than 1 approach (n = 30) had a mean age of 24.2 ± 10.3 years at baseline and were followed for 15.7 ± 12.7 years, having a most recent age of 42.2 ± 17.2 years.

The treatments were as follows: laser and cryotherapy (6 eyes); laser and anti-VEGF (3); radioactive plaque and laser (4); laser, cryotherapy, and PPV (4); cryotherapy and anti-VEGF (3, Fig 2); laser, anti-VEGF, and cryotherapy (3); radioactive plaque, laser, and anti-VEGF (1); cryotherapy, PPV, and anti-VEGF (1); laser and retinopexy (1); cryotherapy and PPV (1); laser, anti-VEGF, and orbital steroid injection (1); laser, anti-VEGF, and intravitreal steroids (1); and cryotherapy, laser, and scleral buckle (1). Ten eyes had serial procedures, up to 13 anti-VEGF injections (mean, 2.8 ± 3.3; median, 2) and 9 sessions of laser (2.1 ± 2.1, median, 1).

Ten eyes (33%) had good response to treatment, with visual acuity maintained and resolved neovascularization, hemorrhages, and RD. Nineteen eyes (63%) had poor response, with decreased vision, development of exudative and/or tractional RD (19 eyes), VH (15 eyes), glaucoma (5 eyes, neovascular and/or open-angle glaucoma), uveitis (2 eyes), retinal and subretinal hemorrhage (2 eyes), and phthisis (3 eyes); and 1 eye (3%) did not have follow-up assessments (Table S2).

#### Good Responders versus Poor Responders

Thirty-five eyes (31%) were good responders, 42 eyes (37%) were poor responders, 22 eyes (19%) had low vision (BCVA > 1.3) at baseline and were only observed, and 12 eyes (11%) did not have longitudinal assessment. Among the good responders, 8 patients were White, 4 patients were Black, and 4 patients were Asian.

Among the eyes with low vision at baseline (n = 39), 22 (56%) were observed with poor response, 10 (26%) had poor response to various treatments, 6 (15%) did not have follow-up assessments, and 1 was observed with good response.

Excluding the patients with low vision at baseline who were observed, those without longitudinal evaluation, and the nonverbal infants, 77 eyes remain. Of these, 43 eyes were treated (56%) and 34 were observed (44%). Twenty-one (62%) observed eyes responded well versus 14 (33%) treated eyes. Dividing the latter into single (n = 14) versus combined (n = 29) treatments, 4 eyes (29%) responded well in the single treatment group (3 laser and 1 cryotherapy), compared with 10 (34%) in the combined subgroup (2 laser and anti-VEGF; 1 laser and brachytherapy; 1 laser, anti-VEGF, and brachytherapy; 1 laser and retinopexy; 1 laser, anti-VEGF, and orbital floor steroids; 1 laser, anti-VEGF, and cryotherapy; 1 laser, cryotherapy, and PPV; 1 anti-VEGF, cryotherapy, and PPV; 1 anti-VEGF and cryotherapy). Six eyes with serial procedures were good responders (43%) compared with 8 with poor response (57%).

Given the multiple combinations and small numbers of each, it is not possible to assess whether one treatment was better than the rest. Although a larger percentage of observed eyes were good responders compared with treated, the severity and nature of the presentation may have been different between groups. Two-thirds of the eyes that responded well to observation had only 1 or 2 quadrants affected compared with one-third of the treated eyes. The milder presentation of the observed eyes may have led to the choice of management and ultimate response.

The phenotypes in the good responders group (n = 35) were 5 eyes from patients with EOSRD, 6 eyes with USH, 1 eye with Hallervorden-Spatz disease, 1 eye with sector RP, and the remaining eyes with RP. Four patients with RP, 2 patients with EOSRD, and 1 patient with CORD did not have follow-up assessments, and the remaining patients with EOSRD, RP, USH, Bardet-Biedl syndrome, achromatopsia, *PAX6*-related dystrophy, CORD, and retinoschisis were in the poor responders group (n = 42).

There was not a significant difference regarding age of CLV onset between good (32.3 ± 18.7) and poor responders (31.2 ± 14.0, *P* = 0.4). The BCVA at CLV onset was significantly better for the good responders (0.5 ± 0.6) than for the poor responders (0.95 ± 0.8, *P* = 0.001). Six eyes in the good responders group had unilateral CLV (17%) versus 8 in the poor responders group (19%). Nine eyes developed exudative RD in the good responders group (26%) versus 35 eyes in the poor responders group (83%). Twenty-one eyes in the good responders group (60%) had CLV changes affecting 1 or 2 quadrants of the retina compared with 17 eyes (40%) in the poor responders group. The time between CLV onset and treatment was not significantly different between groups (1.3 ± 3.0 years for good responders vs. 1.7 ± 6 for poor responders, *P* *=* 0.8).

### Molecular Genetics

**Figure 3:**
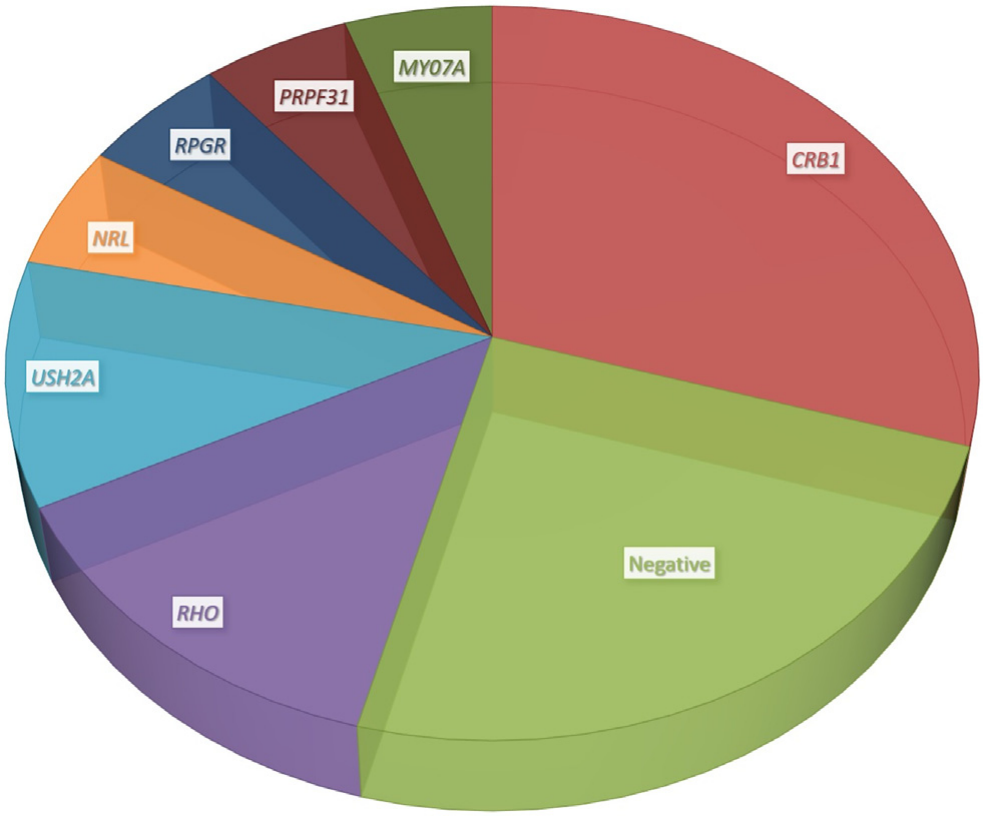
Pie chart representing the most common genes found in the cohort. Fifty-four patients (81%) had genetic testing; 40 had positive results, 9 had negative results, 1 showed inconclusive results, and in 4 patients only the affected gene was recorded, without details of the variant. Eleven patients had disease-causing variants in *CRB1,* 5 in *RHO*, 4 in *USH2A*, and 2 in *PRPF31*, *RPGR*, *NRL*, and *MYO7A*. The remaining genes (*PRPF8, GNAT, GUCA1A, RP1, RS1, PAX6, KLHL7, GUCY2D, FLVCR1, EYS, PANK2, RPE65, CDH3, NR2E3,* and *PDE6B*) were seen in only 1 family each and are not represented in the pie chart. Further information about the variants and demographics can be found in Table S1 (available at www.aaojournal.org).

Fifty-four patients (81%) had genetic testing; 40 had positive results, 9 had negative results, 1 result was inconclusive, and in 4 only the affected gene was recorded, without details of the variant. Eleven patients had disease-causing variants in *CRB1,* 5 in *RHO*, 4 in *USH2A*, 2 in *PRPF31*, *RPGR*, *NRL*, and *MYO7A*, and the remaining genes were seen in only 1 family each (Fig 3, Table S1).

Fifty different variants in 21 genes were present in our cohort; 29 were missense, 8 were nonsense, 5 were splice-site alterations, 5 were small deletions and duplications, 2 were large deletions, and 1 was deep intronic. Forty-one variants (82%) were present in the literature, and 9 variants (18%) were previously unreported (Table S1). Among the latter, 3 were classified as pathogenic, 5 were classified as likely pathogenic, and 1 was classified as a variant of uncertain significance.

The good responders group included eyes from patients with USH1, USH2, *CRB1*-retinal dystrophy, *RHO-*, *USH2A*-, *PDE6B*-, *EYS*-, *PRPF8*-, *RP1*-, *FLVCR1*-, and *PANK2*-RP. Three eyes with *RHO*-RP and 3 eyes with USH2 were poor responders versus 3 and 4 good responders, respectively.

Forty-two eyes with autosomal recessive and X-linked RP and EOSRD had follow-up assessments and did not belong to the group with poor baseline VA that was only observed; 13 eyes corresponded to patients with *CRB1-*retinopathy, and 29 eyes were not *CRB1*-related. Of those caused by variants in *CRB1*, 12 were poor responders (92%) and only 1 was a good responder, whereas among those not *CRB1-*related, 16 were good responders and 13 were poor responders (45%). No significant differences existed between the *CRB1*- and not *CRB1*-related groups regarding age at CLV (26.9 ± 8.7 years versus 30.3 ± 12.5 years, respectively, *P* = 0.45), age at first visit (25.6 ± 10.1 years versus 26.8 ± 10.4 years, respectively, *P* = 0.79), or age at last visit (48.2 ± 14.8 years versus 42 ± 18.5 years, respectively, *P* = 0.42). Patients with autosomal dominant inheritance were excluded from this comparison given that their phenotype can be milder than the other inheritance forms.^23^

## Discussion

This study presents the largest and most diverse cohort of patients with IRD-related CLV to date. The different vascular lesions, management, and longitudinal evaluation are detailed, providing valuable information about the natural history, prognosis, and genetics of this disorder (Table S2).

The prevalence of CLV in this study (0.5%) was less than reported by Khan et al^24^ in 1988 (1.2%–3.6%). This may be associated with the wide availability of multimodal imaging, allowing early diagnosis of IRD and discrimination between differential diagnoses, or to a more thorough understanding and extended sample size of IRD.

Unlike isolated CD,^3^ in IRD-related CLV we found an equal male and female incidence, a minority of patients had unilateral disease (31%), and the mean age of onset was in the fourth decade of life, with a wide range of onset (1–83 years). These characteristics are in keeping with those described in previous small case series and are reinforced in this larger, genetically and phenotypically diverse cohort.^24^^,^^25^

Of note, we saw only 1 affected family member despite 43% of the patients reporting a family history of IRD. It has been postulated that IRD-related CLV may relate to a specific gene(s) or variant(s) that could be inherited, but the fact that it affected only 1 family member makes it a possible IRD complication instead of a unique type of IRD and moreover, that we have such a broad range of underlying genotypes in our cohort.^15^^,^^24^^,^^26^

Fifty-six patients (84%) had mild-moderate visual impairment at baseline and 18 patients (27%) had adult-onset IRD; thus, poor initial VA and early onset do not appear to be necessary risk factors to develop CLV. This was also seen in previous cohorts, in which patients with good initial VA and adult presentations still presented with CLV during follow-up.^15^^,^^18^ Our study has expanded the phenotype of IRD-related CLV, previously restricted to EOSRD and RP, to CORD, macular dystrophy, achromatopsia, and sector RP, indicating that a clinical association (i.e., generalized peripheral retinal vessel thinning) is not evident.

The most common CLV lesion in our cohort was a combination of telangiectasia, RD, and exudates, corresponding to CD stages 2 and 3.^3^^,^^8^ These stages are the most commonly described in IRD-related CLV^14^^,^^27^ and were also the most frequently seen in a large study of isolated CD.^8^ Exudative RD was frequent in this study, affecting 63% of the eyes and mostly the inferior retina. This prevalence and location are consistent with previous reports.^14^^,^^15^^,^^18^ Macular edema or schisis was present in 26% of eyes, a percentage similar to that of patients with IRD without CLV changes.^28^ There is a wide variability in the literature regarding macular cystic changes in CLV, being absent in some case series or present in a smaller, similar, and larger percentage than the one seen in this study.^14^^,^^15^^,^^18^^,^^29^ One quarter of the eyes in this study had VH (27 eyes), a larger subset than in isolated CD.^9^^,^^30^ Shields et al associated the lack of retinal neovascularization with the low incidence of VH in their cohort;^30^ thus, it is possible that the ischemia in IRD-related CLV triggers a different mechanism to cause new vessels and subsequent hemorrhages.^31^

Coats-like vasculopathy is a severe complication of IRD that often decreases visual acuity. This was seen by comparing the baseline VA of patients with and without CLV, by differentiating between those with macular and peripheral CLV, by contrasting VA of the same patients before and after CLV onset, and by noticing a significantly different baseline and latest VA. Patients with CLV in the posterior pole have a worse visual prognosis than those with peripheral lesions. One third of the patients (31%) progressed to more advanced WHO categories of visual impairment over follow-up, and 22% became blind. This percentage is consistent with previous cases of IRD-related CLV,^15^^,^^18^ whereas in isolated CD the condition is mostly unilateral and patients maintain good vision in 1 eye.^29^ The prevalence of glaucoma (20% of eyes) and vasoproliferative tumors was similar to previous reports.^29^^,^^30^^,^^32^

The cohort in this study includes participants diagnosed before intravitreal medications were frequently used. More than half of the eyes (66, 58%) were observed, 22 eyes due to poor initial function. Observation is generally advised in eyes with asymptomatic disease or advanced stage with poor visual prognosis.^8^ Among those with preserved visual function at baseline, 48% responded well to observation, which was a percentage similar to that seen in isolated CD.^9^ The largest percentages of good responders in our cohort were found in the observation and combined therapy groups; therefore, it appears that tailoring the management to the type and location of vascular lesion is key for a favorable prognosis. Current trends show that intravitreal medication may result in a better outcome than cryotherapy or laser alone.^9^^,^^29^ In this study, only 10 patients (15%) received anti-VEGF and 1 patient received a concomitant intravitreal steroid injection, compared with 19 patients (28%) who received cryotherapy and 23 patients (34%) who received photocoagulation. Future work implementing more intravitreal medications in these patients will be useful to determine their suitability in IRD-related CLV.

On the basis of the findings of our cohort, factors possibly predictive of poor response and prognosis include decreased initial VA, development of exudative RD, CLV changes affecting more than 2 quadrants of the retina, and *CRB1-*retinopathy. There were no significant differences regarding age of CLV onset, laterality of disease, time between diagnosis and treatment, or non-White race. In isolated CD, non-White race, diffuse and superior telangiectasias, and exudation have been found to be associated with poor prognosis.^8^

There are several hypotheses explaining why CLV changes occur in IRD, related to hyperpermeable vasodilation, hypoxia secondary to subretinal exudation, and chronic ischemia due to arteriolar attenuation.^15^ A hypoxic state caused by tissue loss and subsequent vascular narrowing also has been described as a possible trigger for CLV.^33^^,^^34^ The appearance of CLV in diverse types of IRD in this cohort somewhat challenges these theories, because the retina functions in nearly opposite ways in RP compared with a macular dystrophy or achromatopsia. An alternative/overlapping explanation is that free radicals produced by misfunctioning retinal cells,^35^ known disruptors of homeostasis and key in the development of DR,^36^ also may play a part in the pathophysiology of IRD-associated CLV. Future possible clinical trials for IRD using antioxidants may be of help in determining whether the incidence of CLV decreases.^37^^,^^38^

### Study Strengths and Limitations

The strengths of this study are the large number of patients with this rare complication of IRD, their diverse phenotype, genotype, and ethnic background, and the multicenter nature. Its limitations include its retrospective nature, incomplete data for some individuals, and nonstandardized methods throughout the centers and periods of time these patients were evaluated. It is also likely that indications for various treatment modalities were not standardized, but therapeutic strategies were a combination of availability as well as physician and patient preference.

## Conclusions

This article describes the largest, most phenotypically and genetically diverse cohort of patients with IRD-related CLV to date. Retinal appearance, disease extent, complications, management, and genotype-phenotype correlations are detailed. Inherited retinal disease–related CLV is a rare complication, affecting less than 1% of patients with IRD. It is sporadic, mostly bilateral, does not have a male predominance, and can occur at any point in the disease, with a mean onset in our cohort in the fourth decade of life. Coats-like vasculopathy can occur in diverse types of IRD and commonly appears as telangiectasia, RD, and exudates. Patients with IRD-related CLV who have decreased initial VA, exudative RD, CLV changes affecting 2 or more retinal quadrants, and *CRB1-*retinopathy may be at greater risk of poor prognosis.
