# Supplementary material for: Coats-like Vasculopathy in Inherited Retinal Disease: Prevalence, Characteristics, Genetics, and Management
Source: Ophthalmology. 2023 Dec;130(12):1327–35. doi: 10.1016/j.ophtha.2023.07.027 (PMC10937259; doi:10.1016/j.ophtha.2023.07.027)
Supplement: Supplementary Table 1 [file mmc1.docx]

| **ID** | **Gender** | **Ethnicity** | **Gene** | **Allele 1** | | **Allele 2** | | **IRD type** |
| --- | --- | --- | --- | --- | --- | --- | --- | --- |
|  |  |  |  | **c.DNA** | **Protein** | **c.DNA** | **Protein** |  |
| 1 | M | White British | NA |  |  |  |  | RP |
| 2 | F | Asian Pakistani | *GNAT2* | c.842_843insTCAG | p.(His282GlnfsTer11) | c.842_843insTCAG | p.(His282GlnfsTer11) | ACHM |
| 3 | M | White British | *GUCA1A* | c.118C>T | p.(Arg40Cys) | c.296A>G | p.(Tyr99Cys) | CORD |
| 4 | F | Asian Pakistani | *CRB1* | c.750T>G | p.(Cys250Trp) | c.750T>G | p.(Cys250Trp) | RP |
| 5 | M | White British | Mit |  |  |  |  | RP |
| 6 | M | NA | *CRB1* | c.2401A>T | p.(Lys801Ter) | c.4094C>A | p.(Ala1365Asp) | EOSRD |
| 7 | F | NA | NA |  |  |  |  | RP |
| 8 | M | White British | *CRB1* | c.2290C>T | p.(Arg764Cys) | c.3879-1203C>G | NA | RP |
| 9 | M | Mixed | *CRB1* | c.3074G>A | p.(Ser1025Asn) | c.3074G>A | p.(Ser1025Asn) | RP |
| 10 | M | White British | *PRPF31* | c.356C>T | p.(Ser119Ter)* [LP] |  |  | ADRP |
| 11 | M | Asian | NEG |  |  |  |  | RP |
| 12 | F | White British | *RHO* | NA |  |  |  | ADRP |
| 13 | F | NA | NEG |  |  |  |  | EOSRD |
| 14 | M | Black | *MYO7A* | c.535_536delAG | p.(Ser179TrpfsTer7)* [LP] | c.535_536delAG | p.(Ser179TrpfsTer7)* [LP] | USH1 |
| 15 | F | Other | *USH2A* | c.13335_13347delinsCTTG | p.(Glu4445_Ser4449delinsAspLeu) | c.10073G>A | p.(Cys3358Tyr) | RP |
| 16 | M | Other | NA |  |  |  |  | RP |
| 17 | F | White British | *PRPF8* | c.7007G>C | p.(Ter2336SerextTer41) |  |  | ADRP |
| 18 | F | Other | USH2A | c.6657+1G>A* [P] | NA | c.6657+1G>A* [P] | NA | USH2 |
| 19 | F | White British | *NRL* | c.148T>A | p.(Ser50Thr) |  |  | ADRP |
| 20 | M | NA | *RPGR* | c.325_326dupTA | p.(Ala110MetfsTer24)* [LP] |  |  | XLRP |
| 21 | F | NA | *CRB1* | c.750T>G | p.(Cys250Trp) | c.750T>G | p.(Cys250Trp) | RP |
| 22 | F | Asian Indian | *RP1* | c.2585C>G | p.(Ser862Ter) |  |  | ADRP |
| 23 | F | NA | NA |  |  |  |  | RP |
| 24 | M | NA | *NRL* | c.148T>A | p.(Ser50Thr) |  |  | ADRP |
| 25 | M | Black | NA |  |  |  |  | RP |
| 26 | M | NA | *CRB1* | c.3520 T>G | p.(Cys1174Gly) | c.2025 G>T | p.(Trp675Cys) | RP |
| 27 | F | White British | NA |  |  |  |  | BBS |
| 28 | M | White British | *CRB1* | c.2843G>A | p.(Cys948Tyr) | c.2668T>A | p.(Cys896Ter) | RP |
| 29 | F | White British | *RHO* | c.1021G>A | p.(Glu341Lys) |  |  | SECTOR RP |
| 30 | F | NA | NA |  |  |  |  | RP |
| 31 | F | White British | *EYS* | c.6137G>A | p.(Trp2046Ter) | c.9131G>T | p.(Trp3044Leu) | RP |
| 32 | F | Black | NEG |  |  |  |  | EOSRD |
| 33 | M | Asian Indian | NEG |  |  |  |  | EOSRD |
| 34 | F | NA | NEG |  |  |  |  | EOSRD |
| 35 | M | Asian Banglasedhi | *USH2A* | c.1546G>A | p.(Gly516Arg) | c.1226G>A | p.(Trp409Ter)* [P] | USH2 |
| 36 | M | NA | *NR2E3* | c.119-2A>C |  | c.932G>A | p.(Arg311Gln) | RP |
| 37 | F | NA | *PRPF31* | c.79G>T | p.(Glu27Ter) |  |  | ADRP |
| 38 | F | White British | *PDE6B* | c.2193+1G>A | NA | c.2193+1G>A | NA | RP |
| 39 | M | White British | *RHO* | c.83A>G | p.(Gln28Arg) |  |  | ADRP |
| 40 | F | Asian Indian | *MYO7A* | c.977 T>A | p.(Leu326Gln) |  |  | USH1 |
| 41 | F | NA | NA |  |  |  |  | RP |
| 42 | M | NA | NA |  |  |  |  | RP |
| 43 | M | Asian Indian | *RPGR* | Deletion of exon 6-11* [LP] |  |  |  | XLRP |
| 44 | F | White British | NA |  |  |  |  | EOSRD |
| 45 | M | Asian Pakistani | *USH2A* | Deletion of exons 50-55 |  | Deletion of exons 50-55 | | USH2 |
| 46 | F | White British | NA |  |  |  |  | USH2 |
| 47 | M | Asian | *KLHL7* | c.422T>C | p.(Val141Ala) |  |  | ADRP |
| 48 | F | Black | NEG |  |  |  |  | RP |
| 49 | M | White British | *GUCY2D* | c.2512C>T | p.(Arg838Cys) |  |  | CORD |
| 50 | F | NA | *RHO* | c.173C>G | p.(Thr58Arg) |  |  | ADRP |
| 51 | F | White British | *FLVCR1* | c.1092+5G>A | NA | c.182C>A | p.(Ser61Ter) | RP |
| 52 | F | Black | NA |  |  |  |  | SECTOR RP |
| 53 | F | White British | *CRB1* | c.584G>T | p.(Cys195Phe) | c.3442T>C | p.(Cys1148Arg) | EOSRD |
| 54 | M | NA | *RS1* | c.574C>T | p.(Pro192Ser) |  |  | XLRS |
| 55 | M | White British | *PAX6* | c.1080C>T | p.(Arg240Ter)* [P] |  |  | *PAX6* -DISEASE |
| 56 | F | White British | NEG |  |  |  |  | EOSRD |
| 57 | F | Asian | *PANK2* | NA |  |  |  | Hallervorden-Spatz |
| 58 | F | White | *CRB1* | c.2843G>A | p.(Cys948Tyr) | c.936T>G | p.(Asn312Lys) | EOSRD |
| 59 | F | White British | NEG |  |  |  |  | SYNDROMIC RP |
| 60 | M | NA | *RPE65* | c.1451G>A | p.(Gly484Asp) | c.1451G>A | p.(Gly484Asp) | EOSRD |
| 61 | M | Asian | *CDH3* | c.325T>G | p.(Trp109Gly)* [VUS] | c.325T>G | p.(Trp109Gly)* [VUS] | EOSRD |
| 62 | M | NA | NA |  |  |  |  | EOSRD |
| 63 | M | NA | NA |  |  |  |  | RP |
| 64 | M | White British | *CRB1* | c.2401A>T | p.(Lys801Ter) | c.3320T>C | p.(Leu1107Pro) | EOSRD |
| 65 | F | Asian | *CRB1* | c.2676+1del | NA | c.2676+1del | NA | EOSRD |
| 66 | F | White | *RHO* | c.759G>T | p.(Met253Ile) |  |  | ADRP |
| 67 | F | White | *USH2A* | c.14218del | p.(Ala4740ProfsTer6)* [LP] | c.14218del | p.(Ala4740ProfsTer6)* [LP] | USH2 |

**Supplementary Table 1:** Demographics and genetic details of the patients of the cohort.

Variants with * are previously unreported and the letter between [] represents the classification. Abbreviations: P: pathogenic; LP: likely pathogenic; VUS: variant of uncertain significance; IRD:

inherited retinal disease; M: male; F: female; NA: not available; NEG: negative; Mit: mitochondrial; RP: retinitis pigmentosa; EOSRD: early onset severe retinal dystrophy; ACHM: achromatopsia; USH: Usher syndrome; BBS: Bardet-Biedl syndrome; XLRS: X-linked retinoschisis; CORD: cone-rod dystrophy; ADRP: autosomal dominant retinitis pigmentosa; XLRP: X-linked retinitis pigmentosa.
