# Supplementary material for: Coats-like Vasculopathy in Inherited Retinal Disease: Prevalence, Characteristics, Genetics, and Management
Source: Ophthalmology. 2023 Dec;130(12):1327–35. doi: 10.1016/j.ophtha.2023.07.027 (PMC10937259; doi:10.1016/j.ophtha.2023.07.027)
Supplement: Supplementary Table 2 [file mmc2.docx]

| **ID** | Coats-like vasculopathy (CLV) | | | | | | **Age at onset** | **Age at CLV** | **Age 1st visit** | BCVA Initial (LogMAR) | | BCVA at CLV (LogMAR) | | Treatment | | | | | **Age last visit** | **FU time** | BCVA Final (LogMAR) | | **Treatment Response** |
| --- | --- | --- | --- | --- | --- | --- | --- | --- | --- | --- | --- | --- | --- | --- | --- | --- | --- | --- | --- | --- | --- | --- | --- |
|  | **Telangiectasia** | **VPT** | **Exudation** | **Ret Hemo** | **Exud RD** | **Quadrants** |  |  |  | **OD** | **OS** | **OD** | **OS** | **Laser** | **Anti-VEGF** | **CT** | **PPV** | **Obs** |  |  | **OD** | **OS** |  |
| 1 OD | 1 |  |  | 1 | 1 | 3 | 61 | 61 | 61 | 1.98 | 0.2 | 1.98 | 0.2 |  |  |  |  | Y | 65 | 4 | 2.28 | 0 | OBSERVED (POOR VA) |
| 2 OD |  |  | 1 | 1 | 1 | 3 | Birth | 23 | 5 | 1.8 | 1.3 | 2.28 | 1 |  |  |  |  | Y | 39 | 34 | 1.5 | 0.9 | OBSERVED (POOR VA) |
| 3 OS | 1 |  |  |  | 1 | 2 | 32 | 44 | 44 | 1 | 1.3 |  |  | Y |  |  |  |  | 66 | 22 | 1.5 | 3 | POOR |
| 4 OD | 1 |  |  |  | 1 | 3 |  | 20 | 13 | 0.2 | 0.2 | 0.2 | 0.3 | Y | Y | Y |  |  | 33 | 20 | 1.4 | 1.4 | POOR |
| 4 OS | 1 |  |  |  | 1 | 3 |  |  |  |  |  |  |  |  |  |  |  | Y |  |  |  |  | POOR |
| 5 OU |  | 2 | 1 |  |  | 3 |  | 42 | 42 | 1 | 1 |  |  |  |  |  |  | Y | 51 | 9 | 1.5 | 1 | POOR-GOOD |
| 6 OD |  |  |  |  | 1 | 3 | Birth |  | 11 | 2.7 | 2.7 |  |  |  |  |  |  | Y |  |  |  |  | NO FU |
| 6 OS |  |  |  |  | 1 | 4 |  |  |  |  |  |  |  |  |  |  |  | Y |  |  |  |  | NO FU |
| 7 OD | 1 |  | 1 |  | 1 | 3 | 15 | 12 | 15 | 2.28 | 0 |  |  | Y | Y |  |  |  |  |  |  |  | NO FU |
| 8 OU | 1 |  |  |  | 2 | 2 | 16 | 25 | 25 | 1 | 1.3 |  |  | Y OS |  | Y OU |  |  | 47 | 22 | 2.7 | 2.7 | POOR-POOR |
| 9 OD | 1 |  |  |  | 1 | 2 | 20 | 36 | 36 | 1.98 | 2.28 |  |  | Y |  | Y | Y |  | 57 | 21 | 2.7 | 2.7 | POOR |
| 9 OS | 1 |  |  |  | 1 | 2 |  |  |  |  |  |  |  |  |  | Y |  |  |  |  |  |  | POOR |
| 10 OD | 1 | 1 |  |  | 1 | 2 | 20 | 45 | 45 | 0.6 | 0.2 |  |  |  | Y | Y |  |  | 70 | 25 | 3 | 0.4 | POOR |
| 10 OS | 1 |  |  |  |  | 3 |  | 45 |  |  |  |  |  |  |  |  |  | Y |  |  |  |  | GOOD |
| 11 OU | 2 |  |  |  | 1 | 3 |  | 31 | 31 | 0 | 0 |  |  |  |  |  |  | Y | 51 | 20 | 3 | 0.6 | POOR-GOOD |
| 12 OD | 1 |  | 1 |  | 1 | 3 | 8 | 9 | 9 | 0.4 | 0.3 |  |  |  | y | Y |  |  | 13 | 4 | 3 | 0.4 | POOR |
| 12 OS | 1 |  | 1 |  | 1 | 2 |  |  |  |  |  |  |  | y | Y |  |  |  |  |  |  |  | GOOD |
| 13 OU |  |  | 2 |  | 1 | 3 | 8 | 14 | 13 | 0 | 0.8 | 0 | 0.8 | Y OU | Y OS | Y OS |  |  | 18 | 5 | 0.2 | 2.28 | GOOD-POOR |
| 14 OU | 1 |  | 1 |  |  | 2 | 4 | 11 | 11 | 0.28 | 0.16 |  |  |  |  |  |  | Y | 19 | 8 | 0.1 | 0.1 | GOOD-GOOD |
| 15 OS |  | 1 | 1 |  |  | 3 | 24 | 37 | 37 | 0.3 | 0.3 |  |  | Y | y |  |  |  | 38 | 1 | 0.4 | 0.6 | GOOD |
| 16 OD | 1 |  | 1 |  |  | 1 | 4 | 9 | 9 | 0.16 | 0.06 |  |  | Y |  |  |  |  | 10 | 1 | 0.1 | 0 | GOOD |
| 17 OS | 1 | 1 |  |  |  | 1 | 8 | 24 | 23 | 1 | 1.98 |  |  |  |  |  |  | Y | 28 | 5 | 0.7 | 1 | GOOD |
| 18 OD |  | 1 |  |  |  | 2 | 32 | 32 | 31 | 1 | 0.6 |  |  | Y |  |  |  |  | 46 | 15 | 2.28 | 3 | POOR |
| 18 OS |  | 1 |  |  | 1 | 4 |  |  |  |  |  |  |  | Y | Y |  |  |  |  |  |  |  | POOR |
| 19 OD | 1 |  |  |  | 1 | 3 | 15 | 29 | 15 | 0.3 | 0.3 |  |  | Y |  | Y | y |  | 54 | 39 | 3 | 3 | POOR |
| 19 OS | 1 |  |  |  | 1 | 2 |  |  |  |  |  |  |  | Y |  | Y |  |  |  |  |  |  | POOR |
| 20 OD | 1 |  |  |  | 1 | 2 |  | 35 | 34 | 0.4 | 0.4 | 1 | 0.4 |  |  |  |  | Y | 41 | 7 | 3 | 1.5 | POOR |
| 20 OS | 1 |  |  |  |  | 3 |  |  |  |  |  |  |  |  |  |  |  | Y |  |  |  |  | POOR |
| 21 OD | 1 |  | 1 |  |  | 2 | 16 | 21 | 20 | 0.3 | 1.98 |  |  |  |  |  |  | Y | 57 | 37 | 2.7 | 3 | POOR |
| 21 OS | 1 | 1 |  |  | 1 | 4 |  |  |  |  |  |  |  |  |  | Y |  |  |  |  |  |  | POOR |
| 22 OU |  |  | 2 | 2 |  | 2 | 59 | 83 | 74 | 0.3 | 0.2 | 2.28 | 0.3 |  |  |  |  | Y | 85 | 11 | 2.7 | 0.2 | OBSERVED (POOR VA)-GOOD |
| 23 OD | 1 |  |  |  | 1 | 3 | 28 | 48 | 36 | 0.6 | 0.6 | 2.28 | 1.5 | Y |  |  |  |  | 68 | 32 | 3 | 2.28 | POOR |
| 24 OD | 1 |  |  |  | 1 | 3 | 47 | 62 | 47 | 0.6 | 0.4 | 2.28 | 0.8 |  |  |  |  | Y | 78 | 31 | 3 | 2.28 | OBSERVED (POOR VA) |
| 24 OS | 1 |  |  |  | 1 | 3 |  |  |  |  |  |  |  |  |  |  |  | Y |  |  |  |  | POOR |
| 25 OS |  |  |  |  | 1 | 3 | 27 | 43 | 27 | 0.3 | 3 | 1 | 3 |  |  |  |  | Y | 58 | 31 | 2.28 | 3 | OBSERVED (POOR VA) |
| 26 OD | 1 |  |  |  |  | 1 | 19 | 42 | 42 | 2.7 | 2.28 |  |  |  |  |  |  | Y | 63 | 21 | 3 | 3 | OBSERVED (POOR VA) |
| 26 OS | 1 |  |  |  | 1 | 3 |  |  |  |  |  |  |  |  |  |  |  | Y |  |  |  |  | OBSERVED (POOR VA) |
| 27 OD | 1 |  |  |  | 1 | 3 | 5 | 12 | 12 | 3 | 2.28 |  |  | Y |  | Y |  |  | 17 | 5 | 3 | 2.7 | POOR |
| 27 OS | 1 |  |  |  | 1 | 2 |  |  |  |  |  |  |  |  |  |  |  | Y |  |  |  |  | OBSERVED (POOR VA) |
| 28 OU | 2 |  |  |  | 2 | 2 | 5 | 40 | 40 | 2.28 | 1 |  |  |  |  |  |  | Y | 50 | 10 | 2.28 | 2.28 | OBSERVED (POOR VA) - POOR |
| 29 OD |  |  | 1 | 1 | 1 | 4 |  | 21 | 19 | 0.2 | 0.2 | 1.5 | 0.2 |  |  |  |  | Y | 37 | 18 | 3 | 0 | OBSERVED (POOR VA) |
| 30 OD | 1 |  | 1 |  | 1 | 4 |  | 19 | 19 | 1.8 | 3 |  |  |  |  |  |  | Y |  |  |  |  | NO FU |
| 30 OS |  |  |  |  | 1 | 2 |  |  |  |  |  |  |  |  |  |  |  | Y |  |  |  |  | NO FU |
| 31 OD | 1 |  |  |  | 1 | 3 | 20 | 37 | 37 | 1 | 2.7 |  |  |  | Y | Y | Y |  | 49 | 12 | 0.8 | 3 | GOOD |
| 31 OS | 1 |  |  |  | 1 | 4 |  |  |  |  |  |  |  |  |  |  |  | Y |  |  |  |  | OBSERVED (POOR VA) |
| 32 OD | 1 |  |  |  | 1 | 3 | 3 | 24 | 24 | 2.7 | 1.8 |  |  |  |  |  |  | Y | 43 | 19 | 3 | 3 | OBSERVED (POOR VA) |
| 32 OS | 1 |  |  |  | 1 | 4 |  |  |  |  |  |  |  |  |  |  |  | Y |  |  |  |  | OBSERVED (POOR VA) |
| 33 OD | 1 |  | 1 |  | 1 | 3 | 0.5 | 1 | 0.5 | 3 | 3 |  |  |  |  |  |  | Y | 10 | 9.5 | 3 | 3 | OBSERVED (POOR VA) |
| 33 OS | 1 |  | 1 |  | 1 | 3 |  |  |  |  |  |  |  |  |  |  |  | Y |  |  |  |  | OBSERVED (POOR VA) |
| 34 OD | 1 |  |  |  |  | 3 | 6 | 46 | 46 | 0.4 | 3 |  |  | Y |  |  |  |  |  |  |  |  | NO FU |
| 34 OS | 1 |  | 1 |  | 1 | 2 |  |  |  |  |  |  |  |  |  |  |  | Y |  |  |  |  | NO FU |
| 35 OD |  |  |  | 1 | 1 | 3 |  | 22 | 22 | 0.6 | 0 |  |  | Y |  | y | Buckle |  | 28 | 6 | 2.28 | 0 | POOR |
| 36 OD | 1 |  |  |  | 1 | 4 |  | 38 | 38 | 0.6 | 0.2 |  |  |  |  | y |  |  | 58 | 20 | 2.28 | 0.4 | POOR |
| 37 OS | 1 |  | 1 |  | 1 | 3 |  | 38 | 21 | 0.4 | 0.4 | 0.8 | 2.28 | Y |  | Y |  |  | 41 | 20 | 1 | 2.28 | POOR |
| 38 OD | 1 |  |  |  |  | 3 | 12 | 34 | 35 | 0.2 | 0.2 |  |  | Y |  | Y |  |  | 72 | 37 | 0.6 | 3 | GOOD |
| 38 OS | 1 |  |  |  |  | 3 |  |  |  |  |  |  |  | Y |  | Y | y |  |  |  |  |  | POOR |
| 39 OS | 1 | 1 | 1 |  |  | 2 | 50 | 61 | 61 | 0 | 0.2 |  |  |  |  |  |  | Y | 82 | 21 | 0.2 | 0.6 | GOOD |
| 40 OD | 1 | 1 | 1 |  | 1 | 3 |  | 30 | 26 | 0 | 0 | 0 | 0 | Y |  | Y | Y |  | 55 | 29 | 0.6 | 0.4 | GOOD |
| 40 OS | 1 |  |  |  | 1 | 3 |  | 31 |  |  |  |  |  | Y |  |  |  |  |  |  |  |  | GOOD |
| 41 OD |  | 1 |  |  | 1 | 2 | 10 | 35 | 35 | 0.3 | 1 |  |  |  |  | Y |  |  | 48 | 13 | 1 | 2.7 | GOOD |
| 41 OS | 1 |  |  |  | 1 | 2 |  |  |  |  |  |  |  |  |  | Y |  |  |  |  |  |  | POOR |
| 42 OU | 2 |  | 2 |  | 2 | 1 | 21 | 31 | 31 | 0.8 | 0.2 |  |  |  |  |  |  | Y |  |  |  |  | NO FU |
| 43 OU | 2 |  |  |  |  | 1 | 16 | 30 | 28 | 0.2 | 0.2 | 0.4 | 0.3 |  |  |  |  | Y |  |  |  |  | NO FU |
| 44 OD | 1 |  |  |  |  | 2 | 10 | 17 | 17 | 0.8 | 0.8 |  |  |  |  |  |  | Y | 20 | 3 | 0.8 | 0.8 | GOOD |
| 44 OS | 1 |  |  |  |  | 3 |  |  |  |  |  |  |  | Y |  |  |  |  |  |  |  |  | GOOD |
| 45 OD | 1 |  |  | 1 | 1 | 1 | 5 | 18 | 18 | 1.98 | 0.2 |  |  |  |  |  |  | Y | 21 | 3 | 3 | 0.2 | OBSERVED (POOR VA) |
| 46 OD | 1 |  |  |  |  | 1 | 21 | 27 | 22 | 0 | 0.2 | 0 | 0 |  |  |  |  | Y | 33 | 11 | 0.2 | 0.2 | GOOD |
| 47 OU | 2 |  |  |  |  | 2 |  | 26 | 26 | 0.2 | 0.2 |  |  |  |  |  |  | Y | 38 | 12 | 0.2 | 0.2 | GOOD-GOOD |
| 48 OU | 1 |  |  |  |  | 3 | 18 | 21 | 21 | 0.8 | 0.6 |  |  |  |  |  |  | Y | 45 | 24 | 1 | 1 | GOOD - GOOD |
| 49 OD | 1 |  | 1 |  | 1 | 1 | 14 | 14 | 14 | 1 | 0.3 |  |  | Y |  |  |  |  |  |  |  |  | NO FU |
| 50 OD | 1 |  |  |  |  | 1 | 24 | 66 | 66 | 0.8 | 0.2 |  |  |  |  |  |  | Y | 76 | 10 | 2.28 | 0.4 | POOR |
| 51 OU | 2 |  |  |  |  | 1 | 11 | 22 | 17 | 0.2 | 0.2 | 0.2 | 0.3 |  |  |  |  | Y | 22 | 5 | 0.2 | 0.3 | GOOD-GOOD |
| 52 OU | 2 |  |  |  |  | 2 | 79 | 83 | 78 | 0 | 0.3 |  |  |  |  |  |  | Y | 90 | 12 | 0.4 | 0.3 | GOOD-GOOD |
| 53 OD | 1 |  | 1 |  |  | 2 | 5 | 40 | 42 | 0.6 | 0.6 |  |  |  |  |  |  | Y | 74 | 32 | 1.8 | 2.28 | POOR |
| 53 OS |  |  |  |  | 1 | 3 |  |  |  |  |  |  |  |  |  |  |  | Y |  |  |  |  | POOR |
| 54 OD | 1 |  |  |  | 1 | 3 |  | 7 | 8 | 1 | 1 |  |  |  |  |  |  | Y | 43 | 35 | 2.28 | 1.5 | POOR |
| 54 OS | 1 |  |  |  | 1 | 4 |  |  |  |  |  |  |  |  |  |  |  | Y |  |  |  |  | POOR |
| 55 OD | 1 |  |  |  | 1 | 3 | Birth | 29 | 29 | 1 | 2.7 |  |  |  |  |  | Drain |  | 47 | 18 | 3 | 3 | POOR |
| 56 OD | 1 |  |  |  | 1 | 3 | Birth | 11 | 12 | 3 | 1.25 |  |  |  |  |  | Y |  | 28 | 16 | 3 | 1.3 | POOR |
| 56 OS | 1 |  |  |  | 1 | 3 |  |  |  |  |  |  |  | Y |  | Y | Y |  |  |  |  |  | POOR |
| 57 OD | 1 |  | 1 |  |  | 3 |  | 10 | 10 | 0.8 | 0.6 |  |  |  |  |  |  | Y | 21 | 11 | 0.6 | 0.6 | GOOD |
| 58 OD | 1 | 1 | 1 |  | 1 | 3 | 3 | 14 | 14 | 0.7 | 0.5 |  |  | Y | Y |  |  |  | 27 | 13 | 0.8 | 3 | GOOD |
| 58 OS | 1 | 1 | 1 |  | 1 | 2 |  |  |  |  |  |  |  | Y | Y |  |  |  |  |  |  |  | POOR |
| 59 OD | 1 |  | 1 |  |  | 2 |  | 7 | 1 | BTL | BTL |  |  | Y |  |  |  |  | 8 | 7 | F&F | F&F | NA |
| 59 OS | 1 |  | 1 |  |  | 2 |  |  |  |  |  |  |  |  |  |  |  | Y |  |  |  |  | NA |
| 60 OD | 1 | 1 | 1 | 1 | 1 | 2 |  | 26 | 19 | 0.6 | 0.8 | 2.7 | 0.6 |  | Y | y |  |  | 26 | 7 | 2.7 | 0.6 | POOR |
| 61 OD |  |  | 1 |  |  | 4 | 1 | 29 | 29 | 2.7 | 2.7 |  |  |  |  |  |  | Y | 37 | 8 | 2.7 | 2.7 | OBSERVED (POOR VA) |
| 61 OS |  |  | 1 |  |  | 4 |  |  |  |  |  |  |  |  |  |  |  | Y |  |  |  |  | OBSERVED (POOR VA) |
| 62 OD | 1 |  | 1 |  | 1 | 4 |  | 57 | 18 | 0.8 | 2.7 |  |  |  |  |  | Y |  | 75 | 57 | 2.7 | 3 | POOR |
| 62 OS |  |  |  |  | 1 | 3 |  |  |  |  |  |  |  |  |  |  |  | Y |  |  |  |  | OBSERVED (POOR VA) |
| 63 OD | 1 |  |  |  |  | 1 |  | 32 | 32 | 0.4 | 0 |  |  |  |  |  |  | Y | 36 | 4 | 0.8 | 0.4 | GOOD |
| 63 OS | 1 |  |  |  |  | 2 |  |  |  |  |  |  |  |  |  |  |  | Y |  |  |  |  | GOOD |
| 64 OD | 1 |  |  |  | 1 | 2 | 1 | 32 | 29 | 1.7 | 1.7 | 1.7 | 1.7 | Y |  | Y |  |  | 43 | 14 | 2.28 | 2.28 | POOR |
| 64 OS | 1 |  |  |  | 1 | 2 |  |  |  |  |  |  |  |  |  |  |  | Y |  |  |  |  | OBSERVED (POOR VA) |
| 65 OU | 2 | 2 | 2 |  | 2 | 2 | 1 | 22 | 5 | 0.7 | 0.6 | 2.28 | 1.5 |  |  |  |  | Y | 24 | 19 | 2.28 | 1.5 | OBSERVED (POOR VA) - OBSERVED (POOR VA) |
| 66 OU | 2 |  | 2 |  | 1 | 4 | 23 | 26 | 26 | 0.15 | 0.3 |  |  | Y |  |  |  |  | 38 | 12 | 1 | 2.28 | GOOD-POOR |
| 67 OU | 2 |  | 2 |  |  | 2 | 13 | 39 | 39 | 1 | 0.7 |  |  | Y |  |  |  |  | 47 | 8 | 1.2 | 1 | GOOD-GOOD |
| **Supplementary Table 2:** Characteristics of the cohort. Fundus, timeline, visual acuity, treatment, and response to treatment. Abbreviations: OD: right eye; OS: left eye; OU: both eyes; Y: yes; N: no; VPT: vasoproliferative tumour; Ret Hemo: retinal hemorrhage; Exud RD: exudative retinal detachment; BCVA: best corrected visual acuity; IRD: inherited retinal disease; CLV: Coats-like vasculopathy; CT: cryotherapy; PPV: pars plana vitrectomy; Obs: observation; FU: follow-up | | | | | | | | | | | | | | | | | | | | | | | |
